# Can Periodical Examinations of Employees Be Useful in Detection of Glycaemia Impairment and Improving Patients’ Adherence to Medical Recommendations?

**DOI:** 10.3390/ijerph15040638

**Published:** 2018-03-30

**Authors:** Andrzej Marcinkiewicz, Wojciech Hanke, Paweł Kałużny, Agnieszka Lipińska-Ojrzanowska, Marta Wiszniewska, Jolanta Walusiak-Skorupa

**Affiliations:** 1 Department of Occupational Diseases and Environmental Health, Nofer Institute of Occupational Medicine, 91-348 Łódź, Poland; agnieszka.lipinska-ojrzanowska@imp.lodz.pl (A.L.-O.); marta.wiszniewska@imp.lodz.pl (M.W.); jolanta.walusiak-skorupa@imp.lodz.pl (J.W.-S.); 2 Department of Environmental Epidemiology, Nofer Institute of Occupational Medicine, 91-348 Łódź, Poland; wojciech.hanke@imp.lodz.pl (W.H.); pawel.kaluzny@imp.lodz.pl (P.K.)

**Keywords:** occupational health, diabetes, glycaemia impairment, impaired fasting glucose, adherence, compliance

## Abstract

Worldwide epidemiological data indicates insufficient diagnosis of diabetes as an increasing public health problem. In the search for solutions to this disadvantageous situation, occupational medicine health services seem to open up a unique opportunity to recognize some abnormalities in the early stages, especially among the asymptomatic working-age population. 316 workers underwent obligatory prophylactic examinations. In patients with twice assayed FGL ≥ 126 mg/dL (7.0 mmol/L) an additional intervention was implemented, including further diagnostic processes and therapy in General Practice (GP), followed by examination by an occupational health specialist within 3 months. The diagnosis of previously unknown diabetes was established among 2.5% of examined workers. All patients referred to the GP due to detected glycaemia impairment visited their doctor and finished the diagnostic process, took up therapy constrained by the occupational health physician to show the effects of intervention within 3 months. Prophylactic medical check-ups allow improved compliance and medical surveillance over glycaemia impairment in patients with prediabetes states, unknown diabetes or uncontrolled clinical course of diabetes. Considering fasting glucose level during mandatory prophylactic examination helps effective prevention of diabetes and its complications and thus provides public health system benefits.

## 1. Introduction

Worldwide epidemiological data indicates insufficient diagnosis of diabetes mellitus (DM) as an increasing problem, as even in developed countries the DM diagnostic rate stands at about 64.2% [1]. Due to the availability of a low-cost screening tool like the measurement of fasting glucose blood level, under-recognizing DM may be interlinked with inconsistencies of the healthcare system, which should provide effective and comprehensive solutions to this issue (in the legal, institutional, organizational, financial and educational aspects). Searching for solutions in this disadvantageous situation, occupational medicine health services seem to offer a unique opportunity to recognize abnormalities in their early stages, especially among the asymptomatic working-age population. About 4 million mandatory employee examinations are carried out yearly in Poland among a population of over 12.5 million in an economically productive age [2]. However, the level of glycaemia in the blood is routinely estimated only in a determined group of workers whose jobs are related to public safety (e.g., drivers) or among workers voluntarily participating in supplementary health preventive programs financed by employers. Mandatory periodical prophylactic examinations may offer a chance for effective prevention of diabetes in the scope of the development of this disease and its complications. Workers’ health checkups are reasonable due to possible detection of prediabetes states (impaired fasting glucose or impaired glucose tolerance), which may be reversible (contrary to diabetes) if effective preventive methods are implemented. Certainly, it is a matter not only of performing a laboratory test, but also of educating a patient in different aspects of a healthy lifestyle and of his adherence to these precautions. Nowadays, a number of terms, e.g., ‘compliance’, ‘adherence’, ‘persistence’, and ‘concordance’, are used to define different aspects of the act of seeking medical attention, acquiring prescriptions and taking medicines appropriately [3]. However, in this aspect adherence, which describes the extent to which a person’s behaviour—taking medication, following a diet, and/or executing lifestyle changes, corresponds with agreed recommendations from a health care provider [3] and seems the most appropriate.

The aim of this study was to evaluate if mandatory periodical prophylactic examinations carried out in Poland may be used to improve the detection of glycaemia impairment among asymptomatic and unaware workers and of their adherence to medical recommendations.

## 2. Materials and Methods

The study group comprised 316 workers referred by various employers to mandatory medical examinations in the period of February–July 2015. Medical check-ups were carried out in the occupational medicine outpatient clinic located in a city in central Poland with a population of 60,000. The criterion for exclusion from the study was previously diagnosed diabetes.

**Figure 1:**
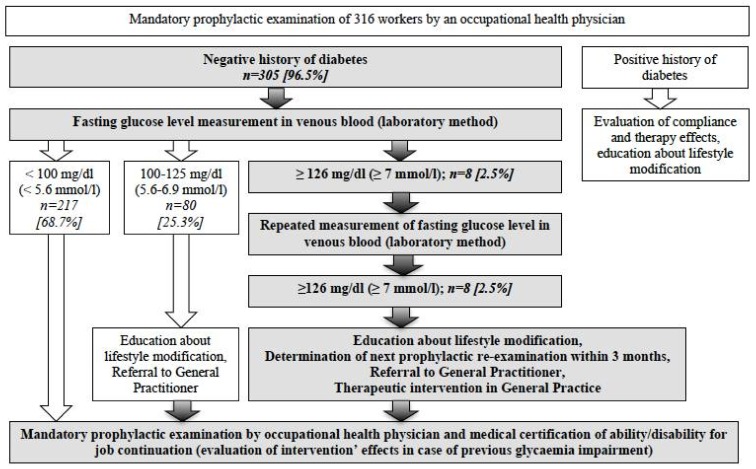
Chart of the study design and intervention implemented among workers with a recognized glycaemia impairment.

Among 305 patients without a previous diagnosis of diabetes, the fasting glucose level (FGL) in venous blood was assayed by a laboratory method. All of the patients with FGL 100–125 mg/dL (5.6–6.9 mmol/L) were educated about the necessity of reducing the modifiable risk factors for DM and further glycaemia control in General Practice (GP). Among patients with FGL > 125 mg/dL (>6.9 mmol/L), another assay was carried out. In case of FGL ≥ 126 mg/dL (7.0 mmol/L) measured in both the 1st and the 2nd assay, that is a criterion qualifying the recognition of diabetes [4], the study intervention procedure was implemented, including referral to a GP for confirmation of the diagnosis and taking up therapy, and the following term of re-examination by an occupational health specialist was established within 3 months. In this period of time, workers with suspected DM had to visit their GPs for further diagnostics. They were then obligated to bring medical information from their GP (about actions taken and their effects) during the next mandatory prophylactic examination carried out in a reduced term-3 months (Figure 1).

Moreover, all of the workers completed a questionnaire of the Findrisc form (which determines of total risk score for developing type 2 diabetes within the next 10 years) [5] and were interviewed about previous glycaemia measurements and further steps in case of detected glycaemia impairment in the past.

As for the characteristics of the study participants the following potential binary factors for glycaemia impairment (based on data from the Findrisc form) were determined: gender, age >45 years, body mass index (BMI) ≥ 25 kg/m^2^, abdominal obesity: waist circumference in men >94 cm and in women >80 cm, low-intensity of usual physical activity (below at least 30 min a day), inappropriate nutrition (unbalanced and poor in vegetables and fruit), family history of DM, detection of glycaemia impairment in the past or lack of prior glycaemia assays.

Statistical analysis applied a crude risk index represented by an odds ratio (OR) of DM incidence in the group with present risk factor of diabetes in relation to the group without this risk factor, together with 95% confidence interval (95% CI). Statistical calculations were conducted using the R software [6].

The study protocol was approved by the local Bioethical Committee at the Nofer Institute of Occupational Medicine in Lodz (decision number 04/2015, 18 February 2015). Participation in the study required an informed written consent.

## 3. Results

**Figure 2:**
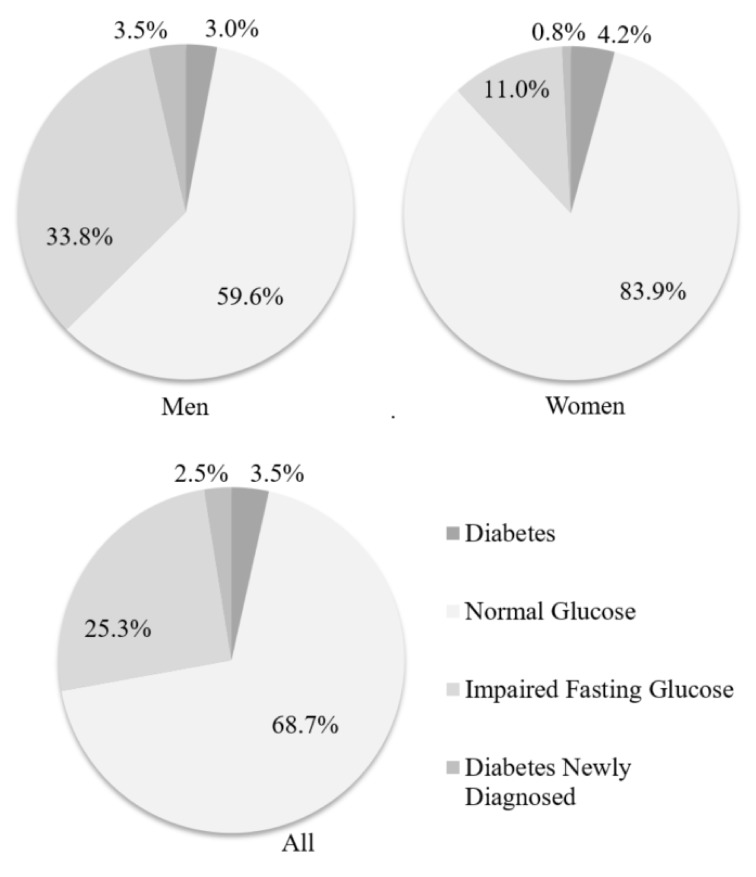
Glycaemia impairment in the study group of workers (*n* = 316).

The study group comprised 316 workers (198 men and 118 women) aged 19–68. The mean age averaged 41.9 ± 11.66 years (mean ± SD) and was higher among male employees (42.6 ± 11.67 years) than female (40.9 ± 11.61 years). Six men (3%) and five women (4.2%) reported suffering from diabetes. In 305 workers without a previous diagnosis of DM, FGL in blood was assayed. In 74 men (37.4%) and 14 women (11.9%) FGL was elevated, among them in 67 males and 12 females FGL ranged 100–125 mg/dL (5.6–6.9 mmol/L) (Figure 2).

In 2.5% of workers who underwent prophylactic examinations previously unknown diabetes was detected (Figure 2): in eight workers (seven men and one woman) in the 1st assay, and then in the 2nd assay as well, FGL exceeded 126 mg/dL (7.0 mmol/L). Due to the established criterion of study intervention, those patients were referred to a GP where finally diabetes was diagnosed in all 8 people and treatment was implemented. This means that 100% of patients have followed medical recommendations.

The most important risk factors of DM in male workers were: overweight, abdominal obesity, low daily vegetable and fruit intake and low usual physical activity. The major pitfalls in female workers were: abdominal obesity, insufficient physical activity and family history of DM (Table 1).

The associations of these factors with the risk of glycaemia impairment is summarized in Table 2. It revealed that most factors were related to hyperglycemia in males and females in similar way. Among lifestyle-related factors in men, the odds for glycaemia impairment were 5.37 times increased in abdominal-obese subjects (95% CI: 2.67–10.82). Remarkably, the odds of glycaemia impairment were not significantly increased (OR 0.99; 95% CI: 0.54–1.84) in the group of men with family history of diabetes, although prevalence of this condition reached 34% (Table 1). The risk of impaired glycaemia was also increased among male workers with overweight (OR 3.41; 95% CI: 1.62–7.15) with low daily physical activity (OR 2.26; 95% CI: 1.25–4.09). Previous episodes of elevated but neglected FGL were strong predictor for impaired glycaemia. Among women, the prevalence of glycaemia impairment was smaller (11%) than among men (33.8%) and therefore the estimates of risk were less reliable.

The odds for glycaemia impairment for females was also increased by abdominal obesity (OR 6.91; CI: 1.47–32.51), overweight (OR 6.34; CI: 1.84–21.89) but unrelated to family history of diabetes (OR 1.74; CI: 0.56–5.38). No significant association between glycaemia impairment and lack of FGL assay in the past, was revealed (OR 0.56 (CI: 0.29–1.06) in men, and 0.36 (CI: 0.07–1.73) in women). Among men glycaemia impairment was also significantly positively associated with age over 45 years and with co-existing arterial hypertension (Table 2).

On the basis of the Findrisc form, 72.5% of the study participants had a low or slightly elevated risk of diabetes type 2 occurrence in the following 10 years. In this group, 37 workers (17%) had IFG and one case of FGL ≥ 126 mg/dL (7.0 mmol/L) was detected. Among other employees with at least a moderate risk of diabetes type 2 occurrence in the following 10 years, seven cases of FGL ≥ 126 mg/dL (7.0 mmol/L) and as a consequence DM were observed, and 43 subjects had IFG (Table 3).

## 4. Discussion

In this study, during mandatory prophylactic examinations, 3.5% of workers declared previous recognition of diabetes, that is less than the estimated prevalence for the Polish population aged 20–79, which is about 7.9% [1]. It is possible that a few patients intentionally dissimulated the disease due to fear of losing the ability to continue their earning job.

Glycaemia impairment was detected in 33.8% of examined men, that is more than the estimated prevalence for prediabetes states, including impaired fasting glucose (IFG) or impaired glucose tolerance (IGT) among Polish males in general (5.8% ± 1.02). An elevated level of fasting glucose was observed in 11% of women, that is similar to the estimated prevalence of prediabetes states among Polish females as a whole (7.25% ± 1.05) [7].

The cause of high prevalence of glycaemia impairment among men may be found in the co-existing typical risk factors for diabetes development, especially more frequent, in comparison with the Polish general population, occurrence of overweight (BMI > 25 kg/m^2^: 71% vs. 61.6%) [8]. In the study group of females, the prevalence of overweight was lower in comparison with Polish general population (approximately 34% vs. 50.3%) [8].

Fasting glucose level in venous blood assay carried out in all workers examined during mandatory prophylactic examinations resulted in a diagnosis of 8 (2.5%) previously unknown cases of DM, which corresponds with the prevalence of unknown diabetes estimated for the Polish general population [1].

It is worth emphasizing that the fasting glucose blood level was measured in venous blood in all of the study participants, and only a positive history of DM constituted an excluding criterion of implementing the intervention. Obligatory glycaemia testing in all employees referred to occupational health physician examinations gave the opportunity to reveal an undiagnosed diabetes with an equal prevalence estimated for the general population. Similarly, Krogsbøll et al., indicated a positive correlation between common health control (not directed on specific abnormalities) and de novo established recognition of health disturbances [9]. However, these researchers also paid attention to a previous lack of data about any impact of these control medical check-ups a decrease in morbidity and mortality. Furthermore, a lot of studies have been published, critically referring to the purpose and effectiveness of general preventive action as part of mandatory examinations of employees [10,11]. On the contrary, the results of our study confirmed the real opportunity for detection of undiagnosed diabetes during prophylactic mandatory periodical examinations, as well as to improve adherence that may reduce both morbidity and mortality. The most important role seems to be played by an appointment of a day of the following mandatory medical check-up in short period of time just after the detection of glycaemia impairment. The pressure put on workers caused by establishing the following examination by the occupational health physician within 3 months resulted in a higher adherence to medical advice (100% of employees took up diagnostics/therapy of DM in GP). Previous literature has suggested that only 36–93% of patients comply with medical advices related to therapy and 10–80% comply with medical advices related to unhealthy lifestyle modification [12].

Among workers, who denied DM recognition in the past, 33% of men and 29% of women admitted to having never had glycaemia testing (comparing with the study carried out by Kobuszewska et al.: 37.7% of males and 17.9% females in the age below 65 years old [13]). The majority of persons with elevated FGL in the past, consulted further diagnostic steps with their GP, however 7% disregarded that result.

Fasting glucose level testing seems to be the most justified in employees subjected to prophylactic examinations who have an increased risk of metabolic disorders. The Findrisc form is a commonly available screening tool for the prediction of DM type 2 development in the near future within 10 years [5,14,15,16]. The results of our study, performed by using the Findrisc form, revealed the distribution of the risk of diabetes in examined workers to be similar to the research conducted by Vandermissen et al., among 275 workers with the average age of 45 years (in our study more patients with a higher risk of DM participated: approximately 14.4% vs. 4.4%) [17]. What is more, contrary to this research [17], not all cases of hyperglycaemia ≥126 mg/dL (7.0 mmol/L) were associated with a moderate or higher risk of DM occurrence (>12 pc on the Findrisc scale). Also, Bergmann et al., indicated towards a lower effectiveness for this tool in the detection of asymptomatic diabetes type 2 [18]. Witte et al., suggested that non-invasive methods of risk assessment requiring further improvement, testing and validation before their implementation as a 1st step in screening diagnostic procedures for diabetes [19].

The study has few shortcomings. The one of them is relatively low number of examined subjects. The only bias we were able to identify is possibly linked to the selection of the population which was done based on schedule provided by employers. However, we do not think it is substantial. We do not know any sources which might possible influence the selection of examined group and to prevent it to be is a good representation of the entire population of workers undergoing preventive examinations in central Poland area.

The strength of the study is proving the potential of employees’ health check-ups for prophylactic purposes. Further research directions should comprise comprehensive examinations in bigger groups as well as follow up to evaluate the results of the intervention.

## 5. Conclusions

It may be worth including fasting glucose blood testing in the scope of prophylactic mandatory examinations of workers due to further health benefits and effective prevention of diabetes that may be also cost-effective for general health preventive services [20]. The obligatory nature of these medical check-ups provides an opportunity for early recognition of undiagnosed diabetes and to improve the patient’s adherence to medical recommendations. A diagnosis of prediabetes states should result in further diagnostic steps, health education and the implementation of preventive activities in the workplace, which are known to be effective [21,22]. Periodical prophylactic examinations of workers, therefore, can successfully fit in the modern model of active diagnosis of asymptomatic population diseases and thus give public health system benefits.

## Figures and Tables

**Table 1:** Characteristics of the study participants without previous recognition of diabetes (*n* = 305).

Risk Factor	Males *n* = 192 (%)	Females *n* = 113 (%)	Both *n* = 305 (%)
Age > 45 years	68 (35)	33 (29)	101 (33)
BMI > 25kg/m^2^	137 (71)	38 (34)	175 (57)
Abdominal obesity: M > 94 cm of WC, F > 80 cm of WC	116 (60)	58 (51)	174 (57)
Low daily physical activity *	81 (42)	57 (50)	138 (45)
Improper diet **	82 (43)	30 (26)	112 (37)
History of arterial hypertension	46 (24)	14 (12)	60 (20)
Family history of diabetes	65 (34)	51 (45)	116 (38)
Positive history of glycaemia impairment	31 (16)	10 (9)	41 (13)
Lack of glycaemia impairment control ***	18 (9)	4 (3)	22 (7)
Lack of previous glycaemia testing	64 (33)	33 (29)	97 (32)

BMI—Body Mass Index; F—Females; M—Males; *n*—number of; WC—waist circumference; * Low daily physical activity—according to Findrisc form [5] lack of at least 30 min of daily physical activity; ** Improper diet—according to Findrisc form [5] diet poor in vegetables and fruit; *** Lack of following control tests despite detection of glycaemia impairment.

**Table 2:** Risk factors for development of the impaired carbohydrate metabolism evaluated in the study group without the prior diagnosis of diabetes (*n* = 305).

Risk Factor	Males (*n* = 192)			Females (*n* = 113)		
	FGL at Reference Range <100 mg/dL *n* = 118 (%)	FGL ≥ 100 mg/dL *n* = 74 (%)	OR (95% CI)	FGL at Reference Range <100 mg/dL *n* = 99 (%)	FGL ≥ 100 mg/dL *n* = 14 (%)	OR (95% CI)
Age > 45 years	24 (20.3)	44 (59.5)	5.74 (3.01–10.95)	26 (26.3)	7 (50)	2.81 (0.9–8.77)
BMI ≥ 25 kg/m^2^	74 (62.7)	63 (85.1)	3.41 (1.62–7.15)	28 (28.3)	10 (71.4)	6.34 (1.84–21.89)
Abdominal obesity *	55 (46.6)	61 (82.4)	5.37 (2.67–10.82)	46 (46.5)	12 (85.2)	6.91 (1.47–32.51)
Low daily physical activity **	41 (34.47)	40 (54.1)	2.26 (1.25–4.09)	46 (46.5)	11 (78.6)	4.22 (1.11–16.07)
Improper diet ***	52 (44.1)	30 (40.5)	0.87 (0.48–1.56)	27 (27.3)	3 (21.4)	0.73 (0.19–2.81)
History of AH	16 (13.6)	30 (40.5)	4.35 (2.15–8.77)	10 (10.1)	4 (28.6)	3.56 (0.94–13.48)
Family history of DM	40 (33.9)	25 (33.8)	0.99 (0.54–1.84)	43 (43.4)	8 (57.1)	1.74 (0.56–5.38)
Positive history of glycaemia impairment	8 (7)	23 (31)	6.20 (2.59–14.81)	5 (5)	5 (36)	10.44 (2.53–43.02)
Lack of glycaemia impairment control ***	4 (3)	14 (19)	6.65 (2.09–21.09)	2 (2)	2 (14)	8.08 (1.04–62.76)
Lack of previous glycaemia testing	45 (38)	19 (26)	0.56 (0.29–1.06)	31 (31)	2 (14)	0.36 (0.07–1.73)

BMI—Body Mass Index. FGL—fasting glucose level. n—number of. * Abdominal obesity—Male >94 cm waist circumference, Female >80 cm waist circumference. ** Low daily physical activity—according to Findrisc form [5] lack of at least 30 min of daily physical activity. ** Improper diet—according to Findrisc form [5] diet poor in vegetables and fruit. *** Lack of following control tests despite detection of glycaemia impairment.

**Table 3:** The risk of developing diabetes type 2 within the next 10 years in patients with detected glycaemia impairment and without a prior diagnosis of diabetes (*n* = 305). According to Findrisc form [5].

10 Years Risk of DM Type 2 Development	All *n* = 305 (%)	Fasting Glucose Level in Venous Blood		
		70–99 mg/dL (3.9–5.5 mmol/L) *n* = 217 (%)	100–125 mg/dL (5.6–6.9 mmol/L) *n* = 80 (%)	≥126 mg/dL (7.0 mmol/L) *n* = 8 (%)
Low (<1%)	141 (46.2)	123 (87.2)	18 (12.8)	0
Slightly elevated (4%)	80 (26.2)	60 (75)	19 (23.8)	1 (1.3)
Moderate (16.6%)	34 (11.1)	16 (47.1)	15 (44.1)	3 (8.8)
High (33%)	44 (14.4)	16 (36.4)	26 (59.1)	2 (4.5)
Very high (50%)	6 (2.0)	2 (33.3)	2 (33.3)	2 (33.3)

DM—diabetes mellitus; Total risk score for developing type 2 diabetes within next 10 years according to Findrisc form [5]: <7 pc—low: estimated 1 in 100 persons will develop DM type 2. 7–11 pc—slightly elevated: estimated 1 in 25 persons will develop DM type 2. 12–14 pc—moderate: estimated 1 in 6 persons will develop DM type 2. 15–20 pc—high: estimated 1 in 3 persons will develop DM type 2 >20 pc—very high: estimated 1 in 2 persons will develop DM type 2.
